# Correlation Analysis of Phenolic Contents and Antioxidation in Yellow- and Black-Seeded *Brassica napus*

**DOI:** 10.3390/molecules23071815

**Published:** 2018-07-21

**Authors:** Yue Wang, Guisheng Meng, Sailing Chen, Yajie Chen, Jinjin Jiang, You-Ping Wang

**Affiliations:** Jiangsu Provincial Key Laboratory of Crop Genetics and Physiology, Yangzhou University, Yangzhou 225009, China; 18252746287@163.com (Y.W.); mengguishengmgs@126.com (G.M.); 18852721748@163.com (S.C.); chenyajie008@163.com (Y.C.)

**Keywords:** *Brassica napus*, yellow seed, phenolic components, flavonoids, antioxidation

## Abstract

*Brassica napus* L. is rich in phenolic components and it has natural antioxidant characteristics which are important to human health. In the present study, the total phenolic and flavonoid contents of developing seeds of yellow- and black-seeded *B. napus* were compared. Both phenolic and flavonoid contents were significantly higher at 5 weeks after flowering (WAF) in black seeds (6.44 ± 0.97 mg EE/g phenolics and 3.78 ± 0.05 mg EE/g flavonoids) than yellow seeds (2.80 ± 0.13 mg/g phenolics and 0.83 ± 0.01 mg/g flavonoids). HPLC–DAD–ESI/MS analysis revealed different content of 56 phenolic components between yellow and black-seeded *B. napus*, including kaempferol-3-*O*-glucoside, isorhamnetin-3-*O*-glucoside, quercetin-3-*O*-sophoroside, procyanidin B2 ([DP 2]), which were significantly reduced in yellow seeds compared with black seeds. Applying the 2,2-diphenyl-1-picrylhydrazyl (DPPH) and 2,2′-azinobis-(3-ethylbenzothiazoline-6-sulfonic acid) (ABTS) radical assay, we found maximum clearance of DPPH and ABTS in the late developmental stages of yellow and black seeds. Additionally, the ferric reducing antioxidant power (FRAP) value maximized at 5 WAF in black seeds (432.52 ± 69.98 μmol Fe (II)/g DW) and 6 WAF in yellow seeds (274.08 ± 2.40 μmol Fe (II)/g DW). Generally, antioxidant ability was significantly reduced in yellow-seeded *B. napus* compared to black rapeseed, and positive correlations between antioxidation and flavonoid content were found in both yellow- and black-seeded *B. napus*.

## 1. Introduction

Rapeseed (*Brassica napus* L.) is the most broadly cultivated *Brassica* species in the world for its high oil (~50%) and protein (~25%) content [1]. As one of the important oil crops for both edible and industrial oil, *B. napus* needs to improve its nutritional value and agronomic yield as the current output is insufficient to meet demand in China [2]. Glucosinolates and sinapate esters are two major anti-nutritional compounds in rapeseed, thus, over the past few decades breeders have put tremendous effort into selecting double-low cultivars (that is, varieties with low-glucosinolate and low-erucic acid) [3]. High concentration of glucosinolates in seeds of *Brassica* crops reduces the nutritional value of seed meal as protein-rich fodder, since their hydrolytic products (e.g., thiocyanate, oxazolidine-2-thiones) interact with the thyroid gland and cause metabolic disturbances [4]. Recently, mutation of genes encoding glucosinolate transporters has reduced the anti-nutritional glucosinolates in *Brassica* oilseeds [5]. Yellow-seeded *B. napus* has been evaluated as having significant advantages over black rapeseed, such as improved nutrients (oil and protein), and reduced anti-nutrients (phenolic compounds, lignin and fiber). These anti-nutrients are not beneficial for oil and seed meal production [6,7,8,9,10]. Hitherto, yellow-seeded *B. napus* were mainly bred by interspecific hybridization of *Brassica* [11], Li et al. first reported yellow rapeseeds from somatic hybridization of *B. napus*-*Sinapis alba* [12]. On the other hand, Brassicaceae crops are well known for their enriched secondary metabolites, especially for phytochemicals with antioxidant activity, including derivatives of hydroxycinnamic acids, sinapic acids, flavonols and anthocyanins [13]. Of these, the accumulation of anthocyanins is responsible for the red, blue, and purple colors in plant species [14]. It has been confirmed that these antioxidant compounds are helpful in preventing cardiovascular, heart disease and cancer by modulating some signaling pathways in mammalian cells [15,16,17,18,19,20]. The medical functions of polyphenols were mainly due to the antioxidant activity, although the mechanism of each polyphenol is not fully understood. In *Aronia prunifolia* hybrids, cyanidin glycosides have been proved to inhibit HeLa human cervical tumor cell proliferation [21]. However, these chemicals greatly reduce the quality of rapeseed oil and meal [22]. Rich phenolics in rapeseeds greatly hinder the use of rapeseed meal for feeding animals since most insoluble flavonoids, especially proanthocyanidins (PAs), can impair the digestibility of seed meal [23]. Phenolic compounds (e.g., sinapoyl esters and PAs) are responsible for the dark color and bitter taste of rapeseed meal and derived protein products, and they are one of the principle factors hampering the use of rapeseeds [13,24]. The breeding of rapeseed with reduced or increased phenolics depends on its main economic use, that is, seed oil/animal fodder or edible vegetable.

The characteristics of yellow-seeded rapeseed are correlated to the variation in phenolic compound synthesis and accumulation [8,25]. The pathways responsible for phenylpropanoid metabolism and flavonoid biosynthesis have been well elucidated in *Arabidopsis* and *Brassica*, providing multiple phenolic compounds (e.g., flavonoid and hydroxycinnamic acid derivatives) [26,27]. Phenolic compounds are difficult to fully identify due to their similar retention time and the limitations of detection technology (e.g., classic high-performance liquid chromatography, HPLC) [23,28]. Yang et al. reported a profile of hydrolysable tannins and other phenolic compounds in emblic leafflower fruits by HPLC-diode array detector (DAD)-electrospray ionization (ESI)-mass spectrometer (MS) analysis [29]. Recently, Shao et al. provided a comprehensive description of phenolic compounds in mature seeds of *B. napus* (black seed) [30]. This study provided the first detailed comparison of phenolic compounds in developing seeds of yellow and black rapeseed via HPLC-photodiode array detector (PDA)-ESI(-)/MS. The yellow rapeseed used in the present study is an introgression line selected from progenies of *B. napus*-*S. alba* somatic hybrids [12]. The black rapeseed is the backcrossing parent used for *B. napus*-*S. alba* hybrids. The antioxidant activity of developing rapeseeds were analyzed and correlated with phenolic content. The comprehensive accumulation pattern of phenolic compounds in developing rapeseeds, accompanied by analysis of the correlation between phenolic content and antioxidant activity, will help to elucidate the character of yellow rapeseeds, the variation in seed color related gene expression, and provide guidance for rapeseed breeding. 

## 2. Results and Discussion

### 2.1. Comparison of Total Phenolic and Flavonoid Content in Developing Seeds of Yellow- and Black-Seeded B. napus

**Figure 1:**
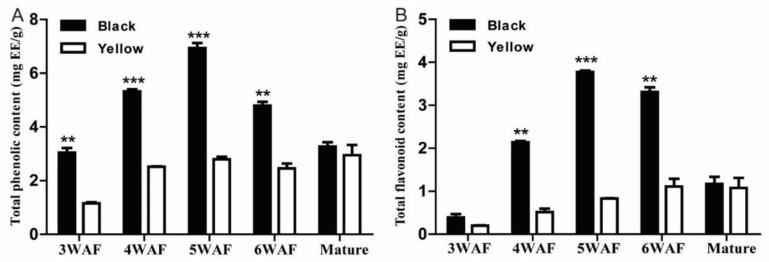
Total phenolic and flavonoid content in developing seeds of yellow- and black-seeded *B. napus*. (**A**). Total phenolic content; (**B**). Total flavonoid content. **, and *** indicate significance at *p* < 0.05, 0.01 and 0.001, respectively. WAF, week after flowering; EE, epicatechin equivalents.

We found that both total phenolic and flavonoid content in black rapeseed maximized at 5 weeks after flowering (WAF) and declined thereafter, whereas, total phenolic and flavonoid content continued to increase as the yellow seeds developed (Figure 1). Also, total phenolic and flavonoid content were significantly higher throughout black seed development (except for mature seeds) than yellow seed. This agrees with the accumulation pattern reported by Jiang et al. [8]. Qu et al. reported that polymeric phenolic compounds started accumulating at 21 days after pollination (DAP), and a significant difference between yellow and black rapeseed was observed from 28 DAP to 49 DAP [25]. 

### 2.2. HPLC–DAD–ESI/MS Analysis of Phenolic Compounds in Yellow- and Black-Seeded B. napus

**Figure 2:**
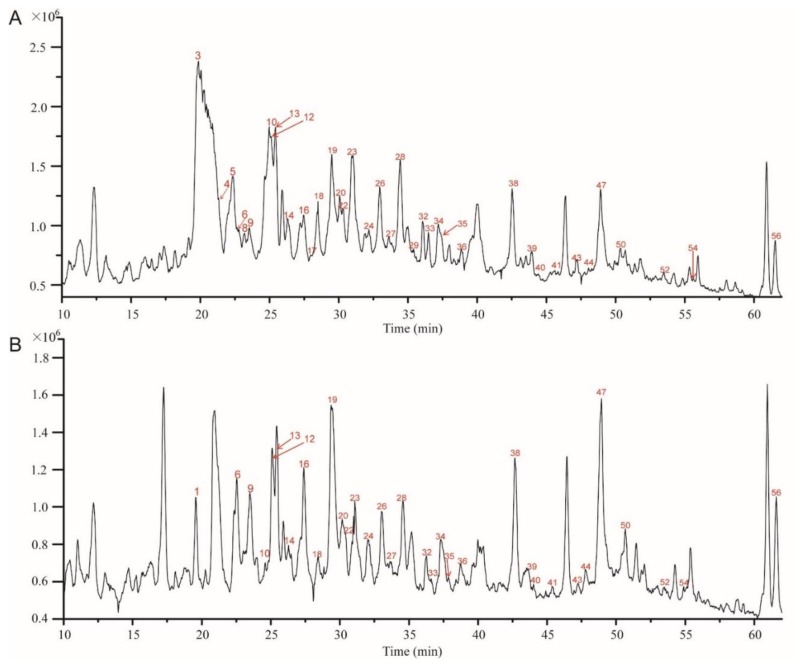
Liquid chromatography-electrospray ionization mass spectrometry (LC-ESI-MS) profile of phenolics from 5 WAF of yellow and black rapeseed. (**A**) black seed; (**B**) yellow seed. Peaks: (1) qn-3-*O*-diglucoside-7-*O*-glucoside; (3) km-3-*O*-sophoroside-7-*O*-glucoside; (4) sinapoylhexose; (5) procyanidin B2 ([DP 2]); (6) km-3-*O*-caffeoylsophoroside-7-*O*-glucoside; (8) qn-3-*O*-sinapoylsophoroside-7-*O*-glucoside; (9) sinapoylhexose**; (10) (-)-epicatechin; (12) km-3-*O*-sinapoylsophorotrioside-7-*O*-glucoside; (13) km-3-*O*-sinapoylsophoroside-7-*O*-glucoside; (14) km-3-*O*-feruloylsophoroside-7-*O*-glucoside; (16) is-3-*O*-glucoside-7-*O*-glucoside; (17) sinapoylhexose***; (18) [DP 3]; (19) km-3-*O*-sophoroside; (20) is-*O*-diglucoside-sulfate; (22) qn-3-*O*-sophoroside; (23) km-3-*O*-diglucoside; (24) *cis*-sinapic acid; (26) is-3-*O*-diglucoside; (27) km-3-*O*-disinapoylgalloyldiglucoside; (28) km-7-*O*-sophoroside; (29) qn-3-*O*-sinapoylsophoroside-7-*O*-glucoside*; (32) km-3-*O*-sinapoyldiglucoside-7-*O*-glucoside; (33) is-3-*O*-sinapoyldiglucoside-7-*O*-glucoside; (34) *trans*-sinapic acid; (35) km-3-*O*-sinapoylsophoroside; (36) [DP 3]*; (38) is-3-*O*-sinapoylglucoside-sulfate-7-*O*-glucoside; (39) km-3-*O*-glucoside; (40) is-3-*O*-glucoside; (41) km-3-*O*-sophoroside-7-*O*-sinapoylglucoside; (43) disinapoylgentiobiose*; (44) is-3-*O*-sinapoylglucoside-7-*O*-glucoside; (47) disinapoylgentiobiose**; (50) putative hydroxycinnamic acid derivative; (52) 1,2,2′-trisinapoylgentiobiose; (54) 1,6-disinapoylglucoside; (56) putative quercetin. km, kaempferol; is, isorhamnetin; qn, quercetin; DP, degree of polymerization of the epicatechin unit. * indicates for different isomers.

**Figure 3:**
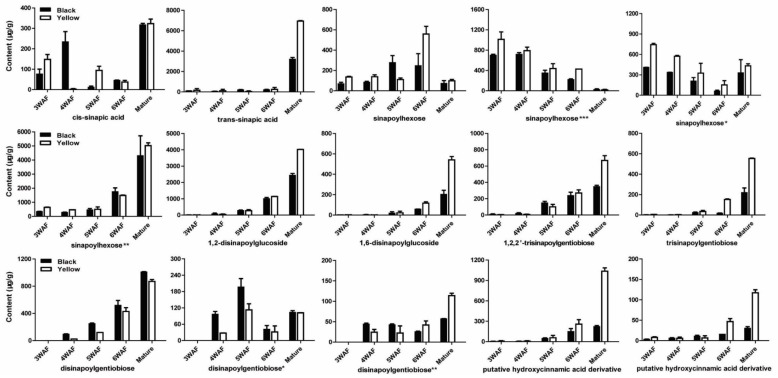
Accumulation pattern of hydroxycinnamic acid derivatives in yellow and black rapeseed. WAF, week after flowering.

Previously, Shao et al. reported a comprehensive polyphenolic profile of *B. napus* [30]. Here, we applied the assay in a detailed comparison of hydroxycinnamic acid derivative and flavonoid content between yellow- and black-seeded *B. napus*. A total of 56 chemical compounds were detected, with several differences found during yellow and black seed development (Figure 2; Supplementary Table S1), including 15 hydroxycinnamic acid derivatives, 21 kaempferols (km), 10 isorhamnetins (is), 5 quercetins (qn), 5 epicatechin and derivatives. We found most of the hydroxycinnamic acid derivatives, including *trans*-sinapic acid, different isomers of sinapoylhexose, disinapoylglucoside, and trisinapoylgentiobiose, were more accumulated in yellow seed compared with black seed. The amount of *cis*-sinapic acid in the early developmental stages of yellow seed was nearly twice of the black seed content, and *trans*-sinapic acid was significantly higher in matured yellow seed than black seed. All the isomers of sinapoylhexose were much more accumulated in the yellow-seeded *B. napus* than the black-seeded *B. napus*. 1,2-disinapoylglucoside, 1,6-disinapoylglucoside, and two isomers of trisinapolygentiobiose accumulated with seed development, which were maximized in mature seeds and were significantly higher in matured yellow seed than black seed. One isomer of disinapoylgentiobiose was more accumulated in matured yellow seed than black seed, whereas another two isomers of disinapoylgentiobiose were less accumulated in yellow seed than black seed. Also, we found two putative hydroxycinnamic acid derivatives were more accumulated at late developmental stages of yellow seed than black seed (Figure 3). 

**Figure 4:**
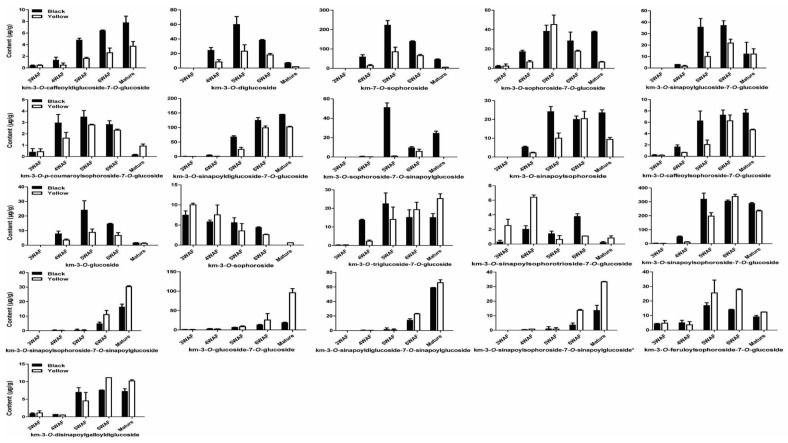
Accumulation pattern of kaempferols in yellow and black rapeseed. km, kaempferol; WAF, week after flowering.

As shown in Figure 4, we found most of the kaempferol derivatives were reduced throughout yellow seed development than black seed, except for km-3-*O*-sophoroside-7-glucoside, km-3-*O*-sophoroside, km-3-*O*-triglucoside-7-*O*-glucoside, km-3-*O*-sinapoylsophorotrioside-7-*O*-glucoside, km-3-*O*-sinapoyldiglucoside-7-*O*-sinapoylglucoside, km-3-*O*-glucoside-7-*O*-glucoside, km-3-*O*-sinapoylsophoroside-7-*O*-sinapoylglucoside, km-3-*O*-sinapoylsophoroside-7-*O*-sinapoylglucoside*, km-3-*O*-feroloylsophoroside-7-*O*-glucoside, km-3-*O*-disinapoylgalloyldiglucoside, which were higher at specific developmental stages of yellow seed compared to black seed. Besides, similar accumulation patterns of kaempferol derivatives were observed in both yellow and black rapeseeds. For instance, km-3-*O*-caffeoyldiglucoside-7-*O*-glucoside accumulated with seed development, km-3-*O*-sophoroside reduced with seed development, whereas km-3-*O*-diglucoside maximized at 5 WAF. 

**Figure 5:**
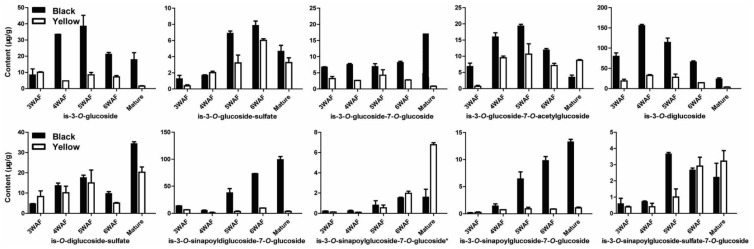
Accumulation pattern of isorhamnetins in yellow and black rapeseed. is, isorhamnetin; WAF, week after flowering.

As illustrated in Figure 5, ten isorhamnetins were shown to have different content in the two rapeseeds, including is-3-*O*-glucoside, is-3-*O*-glucoside-sulfate, is-3-*O*-glucoside-7-*O*-glucoside, is-3-*O*-glucoside-7-*O*-acetylglucoside, is-3-*O*-diglucoside, is-*O*-diglucoside-sulfate, is-3-*O*-sinapoyldiglucoside-7-*O*-glucoside, is-3-*O*-sinapoylglucoside-7-*O*-glucoside which were less accumulated in yellow seed than black seed. However, is-3-*O*-sinapoylglucoside-7-*O*-glucoside* and is-3-*O*-sinapoylglucoside-sulfate-7-*O*-glucoside were more accumulated at later developmental stages of yellow seed than black seed. 

**Figure 6:**
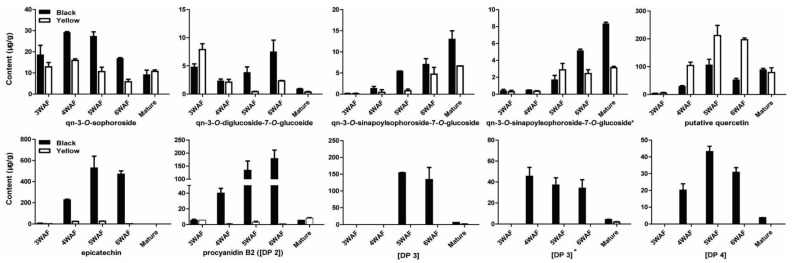
Accumulation pattern of quercetins, epicatechin and derivatives in yellow and black rapeseed. qn, quercetin; DP, degree of polymerization of the epicatechin unit; WAF, week after flowering.

Four quercetins were also reduced in yellow seed compared with black seed, including qn-3-*O*-sophoroside, qn-3-*O*-diglucoside-7-*O*-glucoside, qn-3-*O*-sinapoylsophoroside-7-*O*-glucoside, and qn-3-*O*-sinapoylsophoroside-7-*O*-glucoside*. At 3 WAF, qn-3-*O*-diglucoside-7-*O*-glucoside in yellow rapeseed was higher than black rapeseed (Figure 6). Besides, epicatechin and its four derivatives were significantly reduced throughout yellow seed development, including procyanidin B2 ([DP 2]), [DP 3], [DP 3]*, [DP 4] (Figure 6). 

**Figure 7:**
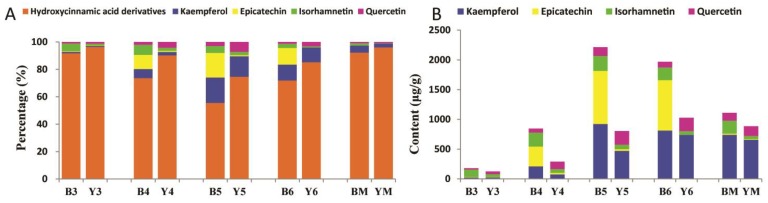
Time-course accumulation pattern of main phenolic components in developing seeds of yellow- and black-seeded *B. napus*. (**A**) Percentage of phenolic components; (**B**) Content of flavonoids.

Previously, Auger et al. identified 13 flavonoids in developing seeds of eight black-seeded *B. napus* genotypes [23]. Jiang et al. reported 19 compounds when comparing polyphenolics in different yellow seed lines and black rapeseed [8]. Qu et al. detected 35 main flavonols in rapeseed and found 2 and 13 unique flavonol derivatives in black and yellow rapeseed, respectively [25]. This is the first comprehensive comparison of flavonoids and hydroxycinnamic acid derivatives between yellow and black rapeseed, which will be helpful in explaining the characteristics of yellow-seeded *B. napus*. We were also able to identify a more detailed accumulation pattern of polyphenolics in the developing seeds of *B. napus*. We found that hydroxycinnamic acid derivatives were significantly higher than flavonoid content, even though flavonoids were more accumulated at 4–6 WAF compared to 3 WAF and the mature stage (Figure 7A). Among the four groups of flavonoids, the amount of kaempferol and epicatechin was higher than isorhamnetin and quercetin (Figure 7B). Epicatechin was mostly accumulated at 4–6 WAF in black rapeseed, but not in yellow rapeseed. The total content of kaempferol in yellow seed was less than black seed, although some of the derivatives with low content were higher at specific stages of yellow seed development compared to black seed. As to quercetin, it was more accumulated in yellow seed after 4 WAF, mainly due to an unknown compound detected with a parent ion at *m*/*z* 301, which has not been reported before. Also, the content of this putative quercetin was much higher than other quercetins, e.g., 106 μg/g and 212 μg/g at 5 WAF for black and yellow seeds, respectively. 

### 2.3. Antioxidant Activity of Yellow- and Black-Seeded B. napus

Different methods were used to assess the antioxidant activity of phenolic compounds (Table 1). The 2,2-diphenyl-1-picrylhydrazyl (DPPH) scavenging activity in yellow rapeseed was significantly lower than black rapeseed throughout seed development except for the mature stage. Similar differences were identified in 2,2′-azinobis-(3-ethylbenzothiazoline-6-sulfonic acid) (ABTS) radical assay. The DPPH radical scavenging ability (RSA) in 4 WAF black seed was 14 times that of yellow seed, and the ABTS radical cation scavenging ability (RCSA) at the same developmental stage was 2.5 times greater in black seed compared with yellow seed. Additionally, different patterns for DPPH and ABTS were observed between yellow and black seeds, which agreed with the phenolic accumulation in the different rapeseeds. As to the ferric reducing antioxidant power (FRAP) assay, we found the polyphenolic extracts from developing black rapeseed exhibited significantly higher reducing power at 3–6 WAF compared with yellow rapeseed. The most significant difference was observed at 4 WAF, 375.38 μM Fe (II)/g DW in black seed and 112.29 μM Fe (II)/g DW in yellow seed. All this data revealed higher antioxidant activity in black rapeseed than yellow rapeseed. Previously, the antioxidant activity and the abundant polyphenolics in *Brassicas,* especially vegetable plants, have been recognized as having putative medical functions in preventing diseases [31,32]. Antioxidant capacity has also been identified in seed meals of *B. juncea* and canola for better extraction of natural antioxidants [33]. This is the first comparison of the antioxidant activity of phenolics from developing seeds of yellow and black rapeseed.

### 2.4. Correlation Analysis of Phenolic Compounds and Antioxidant Activity

Based on the assay of phenolic contents and antioxidant activity of yellow and black rapeseed, correlation coefficients between antioxidant activity and total phenolic/flavonoid content were analyzed (Table 2). We found that the content of phenolics and flavonoids were closely correlated in yellow (0.897) and black rapeseed (0.805), since flavonoid is a major group of phenolics [13]. This is in line with previous reports on lupin and mung beans [16,34]. The three indexes of antioxidant activity were more highly correlated with flavonoid content than phenolic content in the two rapeseed lines, indicating that flavonoids are more related to the antioxidant activity in rapeseed. This agrees with previous reports that *Brassica* vegetables (e.g., broccoli, red cabbage, and brussels sprouts) have the highest antioxidant capability due to high levels of anthocyanins [35]. Farag et al. classified the phenolic metabolites in different organs of *B. napus* and found the antioxidant activity was correlated to some extent with total flavonoid content [28]. Teh et al. reported canola seed cake with higher polyphenols than hemp and flax seed cakes, which was positively correlated with antioxidative capacity [36]. He et al. found the total antioxidant activity in purple Chinese cabbage was highly positively correlated with the anthocyanin content [37]. Thus, the correlation between flavonoids and antioxidant activity in rapeseed is reasonable and could help to explain the different quality of yellow and black rapeseeds. 

## 3. Experimental Section

### 3.1. Plant Materials

Yellow-seeded *B. napus* selected from progenies of somatic hybrids between *B. napus* and *S. alba*, and the black-seeded *B. napus* were cultivated in the experimental field of Yangzhou university. Seeds from each five siliques of five plants were harvested at 3 WAF, 4 WAF, 5 WAF, 6 WAF and mature stage. All the samples were frozen at −80 °C for use.

### 3.2. Extraction of Seed Polyphenols

According to Shao et al. [30], 100 seeds from each developmental stage were dried and weighed for preparing crude extracts of polyphenols. After grinding with 5 mL methanol/acetone/water (40:40:20, *v*/*v*/*v*) for 5 min, samples were sonicated for 15 min and centrifuged at 12,000 rpm for 10 min. The supernatants were collected and combined with supernatants from re-extractions of the pellets. All the crude extracts were concentrated to 1 mL and stored at −80 °C for chemical measurements. 

### 3.3. Quantification of Soluble Phenolic Acids and Flavonoids

Total content of soluble phenolics was measured according to Jiang et al., using the Folin-Ciocalteu method [8]. Epicatechin was used for standard calibration. The total content of soluble flavonoids was measured according to Faudale et al. [38]. The crude polyphenol extract (0.2 mL) was diluted with 2.8 mL ddH_2_O, and mixed with 0.5 mL 5% sodium nitrite, after 6 min, 0.3 mL 10% aluminum chloride was added for 5 min of reaction. Finally, the reaction solution was mixed with 1 mL 1 M NaOH and 0.55 mL ddH_2_O. The absorbance value was recorded at 510 nm using epicatechin as the standard calibration. 

### 3.4. HPLC–DAD–ESI/MS Analysis of Polyphenolic Components

The polyphenol extracts were filtered through 0.45 μm Teflon membrane before HPLC-ESI/MS^2^ analysis, using an Agilent 6460 system (Agilent, Santa Clara, CA, USA) coupled with automatic injector ALS, Quat Pump, 1260 thermostatted column compartment, 1260 DAD detector, and MS QQQ [8]. Generally, 10 μL extracts were separated in a Ultimate XB-C18 column (2.1 × 150 mm, 3.5 μm; Welch, Shanghai, China) accompanied with a C18 precolumn (2 × 4 mm, 3.5 μm; Phenomenex, Torrance, CA, USA). All the settings and elution steps were done as before. After a full scan of all the polyphenols, MS^2^ analysis was performed to confirm chemical structures under a N_2_ gas flow and voltage of 15–40 V. Both positive and negative ion mode and an *m*/*z* range of 100–2000 were selected during ESI source operation. All the analyses was controlled with Agilent MassHunter Workstation Data Acquisition and data analysis was performed using Agilent MassHunter Qualitative Analysis. Confirmation of all the chemicals was based on the UV absorption characteristics, MS^2^ data and rapeseed polyphenol profile reported by Shao et al. [30]. Relative quantification of all the chemicals was based on the ion chromatograms under [M − H]^−^ or [M + H]^+^, using standards (procyanidin B2, (−)-epicatechin, quercetin 3-*O*-β-d-glucoside, and isorhamnetin-3-*O*-glucoside) with similar aglycon cores for calibration.

### 3.5. Antioxidant Activity Analysis

The antioxidant activity of polyphenol extracts was measured using DPPH, ABTS and FRAP methods with few modifications [39,40,41]. Polyphenol extracts (100 µL) were mixed with 3.9 mL 100 µM DPPH reaction solution, and the mixture was allowed 30 min of reaction in darkness and at room temperature. The absorbance was measured at 515 nm against a blank of DPPH solution. The DPPH RSA was calculated as DPPH RSA (%) = (1 − A_515 nm of sample_/A_515 nm of control_) × 100. The reaction of DPPH and 50% methanol was used as control. For ABTS RCSA assay, 0.5 mL polyphenol extract was mixed with 2.5 mL ABTS solution containing 7.4 mM ABTS and 2.6 mM potassium persulfate, and allowed to react for 7 min in darkness and at 30 °C. The absorbance was measured at 734 nm, using ABTS solution without samples as control. The antioxidant ability was calculated as ABTS RCSA (%) = [(A_734 nm of control_ − A_734 nm of sample_)/A_734 nm of control_] × 100. For FRAP assay, 100 µL polyphenol extracts were mixed with 2.4 mL FRAP reagent containing 0.3 M acetate buffer (pH 3.6), 10 mM 2,4,6-tri(2-pyridyl)-s-triazine (TPTZ) solution and 20 mM FeCl_3_ solution (10:1:1, *v*/*v*/*v*). After incubation at 37 °C for 10 min, the absorbance of Fe^2+^-TPTZ was measured at 593 nm against a blank containing ddH_2_O and FRAP reagent. The results were expressed as μM Fe (II)/g DW, in calibration against a standard curve of FeSO_4_ in different concentrations (0.1, 0.2, 0.4, 0.6, 0.8 and 1.0 mM).

### 3.6. Statistics Analysis

All measurements were carried out in triplicate in order to determine reproducibility, and the results are reported as mean ± standard deviation. Correlation analysis of the results were performed with SPSS v.16.0. Statistical significance was defined at level of *p* < 0.05.

## 4. Conclusions

Yellow rapeseed has been a major focus in *B. napus* breeding due to its quality advantages. Molecular analysis of *Arabidopsis* and *Brassica* has proved that this phenotype is correlated to the flavonoid biosynthetic pathway. Phenolic compounds, such as flavonols, anthocyanins, proanthocyanidins, are the main pigments associated with antioxidant activities. Also, this pathway shares common chemical precursors for phenylpropanoid biosynthesis, phenylalanine metabolism, which are related to lignin biosynthesis. Hitherto, comprehensive comparison of phenolic compounds in developing seeds of yellow- and black-seeded *B. napus* has not been reported. Based on HPLC–DAD–ESI/MS analysis, we carried out a comparison of phenolic compounds in yellow and black rapeseeds, involving all of the seed developmental stages. A detailed accumulation pattern of polyphenolics in *B. napus* is also provided. In addition, the antioxidant capability of developing seeds from two rapeseed lines was also detected and correlated with the content of phenolics and flavonoids. Obviously, flavonoid content is significantly correlated with the antioxidant ability of rapeseeds. This more comprehensive profile of phenolic accumulation in yellow and black rapeseeds, should be helpful in breeding rapeseeds with expected antioxidant quality since controversies may exist in breeding rapeseed with higher or lower content of flavonoids, depending on its economic value as seed oil/animal fodder or edible vegetable. With the support of gene expressional data for two rapeseed lines, and the reported expressional profile of flavonoid biosynthesis related genes in Brassicaceae [42], manipulation of genes related to the biosynthesis of specific phenolic compounds could be very helpful in rapeseed breeding.

## Figures and Tables

**Table 1:** Antioxidant activities of black and yellow seeds determined by DPPH, ABTS and FRAP assays.

	DPPH (%)		ABTS (%)		FRAP (μmoL Fe (II)/g DW)	
	Black Seed	Yellow Seed	Black Seed	Yellow Seed	Black Seed	Yellow Seed
**3 WAF**	18.12749 ^a^	1.593625 ^b^	38.36 ^a^	15.36 ^b^	246.48 ^a^	105.32 ^b^
**4 WAF**	52.29084 ^a^	3.685259 ^b^	75.79 ^a^	21.79 ^b^	375.38 ^a^	112.29 ^b^
**5 WAF**	78.18725 ^a^	28.68526 ^b^	98.36 ^a^	54.07 ^b^	432.52 ^a^	236.95 ^b^
**6 WAF**	81.57371 ^a^	41.43426 ^b^	98.36 ^a^	75.50 ^b^	357.46 ^a^	274.08 ^b^
**Mature**	55.07968 ^b^	68.02789 ^a^	95.50 ^a^	98.07 ^a^	330.52 ^a^	313.55 ^b^

Data represent the mean ± standard error (*n* = 3). ^a^ and ^b^ indicate a significant difference at *p* < 0.05 level. WAF, week after flowering.

**Table 2:** Correlation coefficient analysis of phenolic compounds and antioxidant activity in developing seeds of yellow- and black-seeded *B. napus.*

	Flavonoids		ABTS		DPPH		FRAP	
	Black Seed	Yellow Seed	Black Seed	Yellow Seed	Black Seed	Yellow Seed	Black Seed	Yellow Seed
**Phenolics**	0.897 *	0.8053 *	0.535	0.695	0.713	0.607	0.921 *	0.701
**Flavonoids**	--	--	0.747	0.936 *	0.923 *	0.889 *	0.889 *	0.948 *

* indicate significance at *p* < 0.05 level.

## Supplementary Materials

The following are available online, Table S1: List and details of flavonoids and hydroxycinnamic acid derivatives identified in yellow- and black-seeded *B. napus*.
