# Supplementary material for: Correlation Analysis of Phenolic Contents and Antioxidation in Yellow- and Black-Seeded Brassica napus
Source: Molecules. 2018 Jul 21;23(7):1815. doi: 10.3390/molecules23071815 (PMC6100393; doi:10.3390/molecules23071815)
Supplement: Supplementary file 1 [file molecules-23-01815-s001.pdf]

Supplementary Table 1. List and details of flavonoids and hydroxycinnamic acid derivatives identified in yellow- and black-seeded *B. napus*.

Peak	t_R (min)	Measured *m/z* [M-H]$^-$	Calculated formula [M-H]$^-$	Major and important MS^2 ions (*m/z*) (%)	UV λ_{max} (nm)	Tentative identification
1	17.99	787	$C_{33}H_{39}O_{22}$	625(100)	nd	qn-3-*O*-diglucoside-7-*O*-glucoside
2	18.05	933	$C_{39}H_{49}O_{26}$	771(100)	265,345	km-3-*O*-triglucoside-7-*O*-glucoside
3	19.57	771	$C_{33}H_{39}O_{21}$	609(100)	255,350	km-3-*O*-sophoroside-7-*O*-glucoside
4	20.90	385	$C_{17}H_{21}O_{10}$	247(100),223(11),205(6)	240,330	sinapoylhexose
5	22.53	577	$C_{30}H_{25}O_{12}$	425(100),407(44),289(18)	202,280	procyanidin B2 ([DP 2])
6	22.81	933	$C_{42}H_{45}O_{24}$	771(100)	268,330	km-3-*O*-caffeoylsophoroside-7-*O*-glucoside
7	23.23	385	$C_{17}H_{21}O_{10}$	247(100),223(11),205(6)	nd	sinapoylhexose*
8	23.45	993	$C_{44}H_{49}O_{26}$	831(78),625(100)	255,330	qn-3-*O*-sinapoylsophoroside-7-*O*-glucoside
9	24.60	385	$C_{17}H_{21}O_{10}$	247(69),223(100)	nd	sinapoylhexose**
10	24.87	289	$C_{15}H_{13}O_6$	245(100),205(38),179(22)	220,280	(-)-epicatechin
11	25.13	609	$C_{27}H_{29}O_{16}$	489(35),447(100),285(22)	nd	km-3-*O*-glucoside-7-*O*-glucoside
12	25.15	1139	$C_{50}H_{59}O_{30}$	977(100),771(21),609(12),285(15)	270,335	km-3-*O*-s orotrioside-7-*O*-glucoside
13	25.37	977	$C_{44}H_{49}O_{25}$	815(100),609(10)	270,335	km-3-*O*-sinapoylsophoroside-7-*O*-glucoside
14	26.56	947	$C_{43}H_{47}O_{24}$	785(100),609(19)	270,330	km-3-*O*-feruloylsophoroside-7-*O*-glucoside
15	27.62	917	$C_{42}H_{45}O_{23}$	755(100)	nd	km-3-*O*-*p*-coumaroylsophoroside-7-*O*-glucoside
16	27.80	639	$C_{28}H_{31}O_{17}$	477(100),315(21)	nd	is-3-*O*-glucoside-7-*O*-glucoside
17	28.57	385	$C_{17}H_{21}O_{10}$	325(69),295(100),265(81),223(24)	240,325	sinapoylhexose***
18	28.70	865	$C_{45}H_{37}O_{18}$	577(62),451(71),407(100),289(78)	240,280	[DP 3]
19	29.56	609	$C_{27}H_{29}O_{16}$	447(100),429(31)	nd	km-3-*O*-sophoroside
20	30.22	719	$C_{28}H_{31}O_{20}S$	639(100),403(27),315(12)	nd	is-*O*-diglucoside-sulfate
21	30.466	1153	$C_{60}H_{49}O_{24}$	865(43),575(100),413(35),289(42)	nd	[DP 4]
22	30.80	625	$C_{27}H_{29}O_{17}$	463(100),445(44),300(81)	265,345	qn-3-*O*-sophoroside

23	31.13	609	$C_{27}H_{29}O_{16}$	447(53),285(100)	265,335	km-3-*O*-diglucoside
24	32.43	223	$C_{11}H_{11}O_5$	193(100),164(74)	nd	*cis*-sinapic acid
25	32.83	933	$C_{42}H_{45}O_{24}$	771(100),609(10)	nd	km-3-*O*-caffeoyldiglucoside-7-*O*-glucoside
26	32.98	639	$C_{28}H_{31}O_{17}$	477(45),315(100),313(46)	256,350	is-3-*O*-diglucoside
27	33.69	1173	$C_{56}H_{53}O_{28}$	815(100)	275,330	km-3-*O*-disinapoylgalloyldiglucoside
28	34.48	609	$C_{27}H_{29}O_{16}$	447(9),429(100),285(91)	nd	km-7-*O*-sophoroside
29	35.43	993	$C_{44}H_{49}O_{26}$	831(100)	265,330	qn-3-*O*-sinapoylsophoroside-7-*O*-glucoside*
30	35.61	681	$C_{30}H_{33}O_{18}$	476(100),315(29)	nd	is-3-*O*-glucoside-7-*O*-acetylglucoside
31	35.83	557	$C_{22}H_{21}O_{15}S$	477(100),395(7),315(18)	265,320	is-*O*-glucoside-sulfate
32	36.16	977	$C_{44}H_{49}O_{25}$	815(100),653(28),609(4)	265,330	km-3-*O*-sinapoyldiglucoside-7-*O*-glucoside
33	36.53	1007	$C_{45}H_{51}O_{26}$	845(100)	265,330	is-3-*O*-sinapoyldiglucoside-7-*O*-glucoside
34	37.13	223	$C_{11}H_{11}O_5$	208(100),179(32),164(51)	nd	*trans*-sinapic acid
35	37.50	815	$C_{38}H_{39}O_{20}$	623(66),609(100),591(34)	nd	km-3-*O*-sinapoylsophoroside
36	39.00	865	$C_{45}H_{37}O_{18}$	695(37),587(76),407(84),289(100)	nd	[DP 3]*
37	43.15	753	$C_{34}H_{41}O_{19}$	529(100),487(42)	nd	disinapoylgentiobiose
38	43.59	925	$C_{39}H_{41}O_{24}S$	763(100)	275,330	is-3-*O*-sinapoylglucoside-sulfate-7-*O*-glucoside
39	43.79	447	$C_{21}H_{19}O_{11}$	357(23),285(100),284(80)	265,345	km-3-*O*-glucoside
40	44.10	477	$C_{22}H_{21}O_{12}$	315(43),314(100)	265,355	is-3-*O*-glucoside
41	44.43	977	$C_{44}H_{49}O_{25}$	609(100)	265,340	km-3-*O*-sophoroside-7-*O*-sinapoylglucoside
42	45.64	1183	$C_{55}H_{59}O_{29}$	977(15),815(100),609(21),285(7)	270,330	km-3-*O*-sinapoylsophoroside-7-*O*-sinapoylglucoside
43	47.23	753	$C_{34}H_{41}O_{19}$	529(100)	nd	disinapoylgentiobiose*
44	47.42	845	$C_{39}H_{41}O_{21}$	683(100),477(21),357(7)	270,330	is-3-*O*-sinapoylglucoside-7-*O*-glucoside
45	48.46	1183	$C_{55}H_{59}O_{29}$	815(100),623(37)	270,330	km-3-*O*-sinapoyldiglucoside-7-*O*-sinapoylglucoside
46	48.81	845	$C_{37}H_{41}O_{17}$	683(100),477(21),357(7)	nd	is-3-*O*-sinapoylglucoside-7-*O*-glucoside*
47	49.01	753	$C_{34}H_{41}O_{19}$	529(100)	nd	disinapoylgentiobiose**
48	49.15	1183	$C_{55}H_{59}O_{29}$	815(80),609(100)	nd	km-3-*O*-sinapoylsophoroside-7-*O*-sinapoylglucoside*
49	49.21	815	$C_{38}H_{39}O_{20}$	653(100),447(11),284(4)	265,330	km-3-*O*-sinapoylglucoside-7-*O*-glucoside

50	49.961	757	$C_{37}H_{41}O_{17}$	595(100),223(47)	nd	putative hydroxycinnamic acid derivative
51	50.62	591	$C_{28}H_{31}O_{14}$	367(100),223(91)	245,320	1,2-disinapoylglucoside
52	52	959	$C_{45}H_{51}O_{23}$	735(100),529(8),511(11)	nd	1,2,2´-trisinapoylgentiobiose
53	54.61	757	$C_{37}H_{41}O_{17}$	561(100),337(49),223(19)	nd	putative hydroxycinnamic acid derivative
54	55.70	591	$C_{28}H_{31}O_{14}$	531(33),367(100),205(31)	235,330	1,6-disinapoylglucoside
55	57.59	959	$C_{45}H_{51}O_{23}$	735(100)	nd	trisinapoylgentiobiose
56	61.65	301	$C_{15}H_{10}O_{7}$	301(100)	nd	putative quercetin

Abbreviations: km, kaempferol; is, isorhamnetin; qn, quercetin; DP, degree of polymerization of the epicatechin unit. nd = not detected. * indicates for different isomers.
